# Trends in admissions to a child and adolescent neuropsychiatric inpatient unit in the 2007–2017 decade: how contemporary neuropsychiatry is changing in Northwestern Italy

**DOI:** 10.1007/s00787-021-01794-7

**Published:** 2021-04-29

**Authors:** Federico Amianto, Luca Arletti, Chiara Baietto, Chiara Davico, Giuseppe Migliaretti, Benedetto Vitiello

**Affiliations:** 1 Neurosciences Department, Psychiatry Section, Service for Eating Disorders, University of Torino, Via Cherasco 11, 10126 Torino, Italy; 2 Department of Public Health and Pediatric Sciences, Section of Child and Adolescent Neuropsychiatry, University of Turin, Turin, Italy; 3 Department of Pediatrics and Pediatric Sciences, Regina Margherita Pediatric Hospital, Turin, Italy; 4 Department of Public Health and Pediatric Sciences, Section of Statistics, University of Turin, Turin, Italy

**Keywords:** Adolescence, Psychopathology, Depression, Healthcare system, Annual Percent Change (APC)

## Abstract

**Purpose:**

Rising levels of psychopathology in the adolescent population have been evidenced in the last few years throughout the Western world. We aim to examine how contemporary neuropsychiatry is changing in Northwestern Italy and how this impacts inpatient services.

**Methods:**

The present research considered the 1177 admissions to a public neuropsychiatric inpatient service in the 2007–2017 decade. The annual percentual change (APC) was analysed for the total admissions, the number of the neurological vs psychiatric admissions, the length of inpatient stay, and the mean age at admission, also accounting for sex differences. The annual trend was also calculated for each diagnosis.

**Results:**

The overall number of inpatient admissions decreased significantly (APC = − 5.91), in particular for children under 12 years of age (APC = − 7.23). The rate of neurologic diagnoses significantly decreased (APC = − 26.44), while the length of the inpatient stay (APC = 6.98) and the mean age at admission (APC = 6.69) increased. Among the psychiatric diagnoses, depression significantly rose (APC = 41.89), in particular among female adolescents (APC = 40.30).

**Conclusions:**

These data document a substantial change in the utilization of inpatient neuropsychiatric services for children and adolescents, with a major increase in psychiatric hospitalizations and a parallel decrease in neurological ones. These trends call for greater attention to early preventive intervention in mental healthcare system.

## Introduction

Psychopathological problems of the teenagers are growing in Italy as well as in the rest of Western world [1, 2]. A number of reports indicate that teenagers display worldwide increasing rates of conduct problems and other externalizing disturbances, such as abuse of drugs or alcohol [3, 4], depression [5–7], self-injurious or life-threatening behaviour [8]. Despite their incidence appear unchanged, also the prevalence of eating disorders is increasing in childhood and adolescence due to the long duration of illness [9].

The increase of adolescence psychopathology is crucial because it impairs the current social and relational functioning of the youth with possible consequences for his/her family adjustment, personality development, and academic career [2]. It may represent a concrete risk for long-lasting impairment of mental and also physical health in adulthood [3]. Internalizing and externalizing disorders in adolescence may often produce vicious circles with serious consequences. For instance, the reduction of sleep caused by internet addiction, substance abuse or alcohol can cause deterioration of social relations, conduct disorders, anxiety disorders or major depression [10], increasing also the suicidal ideation [11]. Research also supports the association between the abuse of cannabis or other "light" drugs and the development of psychotic disorders such as schizophrenia [12]. Moreover, the emotional and relational stress in adolescence may cause an increase in BMI which is related to the development of obesity in adulthood [13]. Last but not least, bullying is linked to abnormalities in personality development, to syndromes such as ADHD, to issues of affective-relational development [14], and it can be a cause of suicide [15].

The psychopathological expressions of the adolescents require specific therapeutic and management approaches which differ from those of early childhood psychiatry. Often the adolescents display physical characteristics which are similar to those of the adults and their psychopathological expressions are often characterized by self-harming or violent behaviours [2]. It is for this reason that despite the existence of effective therapies the use of coercive means is still a relevant problem in adolescents’ management [16].

Frequently, the increase in the use of emergency services to manage the adolescents with a mental disorder is also consequent to the sense of impotence of the family in containing the adolescent offspring. The contemporary changes in social and relational dynamics leading to a reduction of the role of authority in the familial relationships may have contributed to contemporary inability of parents in establishing relations of authority and hierarchy to contain the adolescent on the affective and regulatory side of the parenting relationship [17].

Current literature evidences that the parents are often thwarted to build and manage a relationship with their children also because personal problems [18]. Sometimes the family is also the bearer of psychopathology through physical or psychological abuse, neglect and parenting inability [19, 20]. Family conflicts, distortion of communication, and intra-family stress predispose to internet addiction [21]. A lack in parenting skills is also linked to somatization [22], eating disorders [17] and borderline personality [23].

Clinicians perception support rise in psychopathological diagnoses and inpatients admissions among adolescents [1]. At the best of our knowledge no study reports consistent data confirming this trend in the last decade. Data reporting about large cohort studies have been recently published to describe the rising of adolescents’ problems in the last decade [5–7], nevertheless at the best of our knowledge no study reported the description of inpatient hospital treatments in the same period.

The present paper reports the picture of 11 years of observation of the inpatient admissions into a Neuropsychiatric Inpatient Service of an Italian paediatric hospital showing the trend of inpatient admissions with respect to the sex and age of admission, diagnosis and the length of inpatient stay. A better insight into this phenomenon may be useful for planning future organization of child and adolescent therapeutic services and also to adopt prevention strategies involving paediatricians, families and child and adolescent psychiatrists.

## Methods

All the admissions to the Neuropsychiatric Inpatient Service of the Regina Margherita Paediatric Hospital (OIRM) in Turin over the eleven years 2007–2017 were examined. This service has a function of hub for the northwest region of Italy called “Piedmont and Valle D’Aosta” (PIVA), where it has been the only neuropsychiatric inpatient service through 2017. The region includes urban, industrial, and rural areas, and is home to about 4.5 million people.

The available data consisted in: diagnosis at discharge, patient age and gender at admission, number of admissions for each month of the year, and length of hospitalization.

Inclusion criteria: they were considered in the data elaboration 1. all the hospital admissions with a minimal duration of 2 days (48 h); 2. the admissions for which the neuropsychiatric diagnosis at admission was confirmed at discharge. The 48 h stay for admissions is the time-limit for the administration of the hospital for asking reimbursement to health authorities. Admissions with shorter duration are invalidated.

### Admission rules and policy

During the considered decade the neuropsychiatric inpatient service of the Regina Margherita Paediatric Hospital (OIRM) was the only emergency centre for child neuropsychiatry in the entire PIVA region. So hospital admissions were regulated with an absolute priority for those from the emergency room and a relative priority for those from the hospital's paediatric wards who temporarily admitted neuropsychiatric patients from the emergency room  who found no place in the neuropsychiatric department. All other elective admissions agreed with the territorial services had a lower priority. The present study included all the patients admitted to the inpatient service from any of these possible sources. The decision of admission was entirely based on the severity of the disorder and the impossibility of the other services (i.e. territorial neuropsychiatric and paediatric services) to manage it out of the inpatient service. All admitted patients access into the hospital through the Emergency Service where the degree of severity of their disease is evaluated by the neuropsychiatric emergency doctor, so the criteria for inpatient admission are homogeneous. This is why the number of hospitalizations in the neuropsychiatric hospital service is an indicator of the need for emergency neuropsychiatric hospitalizations in the region.

### Ethics

The study consisted in analyses of clinical and administrative data that had been collected as part of routine practice. The privacy of the sensitive data was protected during the analyses.

### Statistical analysis

The age-standardized incidence rate (hospitalizations per 100,000 inhabitants per year) and the relative 95% confidence interval (95% CI) was estimated for each diagnostic category overall and separately by sex (per 100.000). Population data between 2007 and 2017 were obtained from census data and annual inter-census estimates provided by the Evolutionary Demographic Database of the Piedmont Region [24]^.^ Time-trend analysis was based on annual incidence rates by age groups (children from 0 to 11 years old; adolescents from 12 years old to 18 years old). The trend over time was estimated as the Annual Percent Change (APC) and relative 95% CI in the incidence rate using Poisson regression analysis separately by sex and adjusted for age [25]. All the statistics were performed using SAS Statistic Software ®.

## Results

### Sample description

A total of 1177 inpatient admissions were considered in the final analysis. The age at admission ranged from less than one year to 17 years.

Overall there were 261 ICD-9 primary diagnoses, which were grouped into 26 diagnostic groups according to ICD-11 classification. We included in a larger group all diagnoses with less than five cases in the considered 11 years (e.g. we included in the diagnosis of depression also four diagnoses of adjustment disorders with depressed mood; in the diagnosis of epilepsy all the subtypes of idiopathic epilepsy).

The total number of hospitalizations with a psychiatric diagnosis was 871, subdivided into the following 15 diagnostic groups: Eating Disorders (*n* = 238), Disruptive Behaviour Disorders (*n* = 114), Somatoform Disorders (*n* = 103), Psychotic Disorders (*n* = 97), Personality Disorders (*n* = 84), Major Depression (*n* = 63), Self-poisoning (*n* = 45), Autism (*n* = 23), Obsessive–Compulsive Disorders (*n* = 18), Adjustment Disorders (*n* = 15), Post-Traumatic Stress Disorder (*n* = 14), Phobias (*n* = 12), Tourette Syndrome (*n* = 12), Panic Attack and Agoraphobia (*n* = 8), Substance Abuse (*n* = 6), Maltreatment (*n* = 6), residual psychiatric diagnoses (*n* = 13).

The total number of hospitalizations with a neurological diagnosis was 273, subdivided into the following 11 diagnostic groups: PNS Injuries (*n* = 16), Epilepsy (*n* = 161), CNS Injuries (*n* = 38), Neurodevelopment Disorders (*n* = 18), Traumatic Injuries (*n* = 13), Visual System Disorders (*n* = 8), Muscle-skeletal Disorders (*n* = 8), Headache (*n* = 6), Rheumatic Chorea (*n* = 5). Thirthy-three patients were excluded from the analysis for missing data.

Table 1 sums up the data concerning the days of inpatient stay, mean age, gender distribution, and total psychiatric vs neurological diagnosis at admission that have been collected and analysed year by year.

**Table 1:** Days of inpatient stay, mean age, gender distribution and comparison of psychiatric vs neurological diagnoses at admission year by year

Year	Inpatient days	Age	Sex		Psychiatric	Neurologic
	[mn ± sd]	[mn ± sd]	Male *n* (%)	Female *n* (%)	*n* (%)	*n* (%)
2007	34.07 ± 32.19	8.47 ± 5.49	76 (50%)	76 (50%)	91 (63%)	53 (37%)
2008	32.00 ± 34.51	7.72 ± 5.43	69 (48%)	74 (52%)	69 (50%)	69 (50%)
2009	44.29 ± 40.29	8.70 ± 5.60	47 (37%)	79 (63%)	73 (61%)	47 (39%)
2010	41.77 ± 35.44	9.68 ± 5.96	51 (46%)	59 (54%)	71 (68%)	34 (32%)
2011	48.60 ± 42.96	10.83 ± 4.65	37 (40%)	55 (60%)	64 (72%)	25 (28%)
2012	58.70 ± 43.30	11.51 ± 4.39	38 (43%)	50 (57%)	73 (84%)	14 (16%)
2013	61.80 ± 44.33	11.66 ± 3.93	28 (31%)	63 (69%)	78 (90%)	9 (10%)
2014	71.33 ± 46.51	12.64 ± 3.29	12 (16%)	64 (84%)	74 (97%)	2 (3%)
2015	63.57 ± 55.00	12.21 ± 3.22	39 (27%)	65 (63%)	90 (87%)	14 (13%)
2016	70.57 ± 44.46	13.16 ± 2.50	21 (15%)	62 (85%)	80 (98%)	2 (2%)
2017	49.73 ± 32.47	14.36 ± 2.42	22 (20%)	90 (80%)	108 (97%)	3 (3%)

*mn* mean; *sd* standard deviation; *n* number of patients

### Trends in hospitalization incidence and patient demographics,

Table 2 presents the main results of the trend analysis in the period 2007–2017 discussed in the following paragraphs, relating to sex and age of the patient, number of hospitalization days and diagnosis. Results are showed as total and separately for children and adolescents. Trend analysis of the admission number (APC = − 5.91), shows a significant decrease of the percentage of males (APC = − 13.97) compared to a substantial stability of the number of female admissions (APC = − 0.79). Male children display a significant reduction of inpatients admissions (APC = − 7.40), while female adolescents a significant increase (APC = 5.69).

**Table 2:** APC of Inpatient Admissions characteristics

Variables	Total				Females				Males			
	APC	95% CI inf	95% CI sup	*p*	APC	95% CI inf	95% CI sup	*p*	APC	95% CI inf	95% CI sup	*p*
Number of inpatient admissions												
All	− 5.91	− 10.20	− 1.42	0.011	− 0.79	− 4.78	3.36	0.703	− 13.97	− 18.74	− 8.92	0.000
Children	− 7.23	− 12.18	− 1.99	0.007	− 3.22	− 7.06	0.78	0.113	− 7.40	− 11.80	− 2.78	0.002
Adolescent	3.45	− 2.74	10.02	0.281	5.67	0.70	10.90	0.025	− 2.61	− 7.55	2.59	0.319
Neurologic admissions												
All	− 26.44	− 34.14	− 17.84	0.000	− 24.54	− 32.41	− 15.75	0.000	− 26.21	− 34.03	− 17.47	0.000
Children	− 11.10	− 18.40	− 3.15	0.007	− 6.23	− 11.89	− 0.20	0.043	− 11.05	− 17.62	− 3.95	0.003
Adolescent	2.49	− 7.13	13.11	0.624	3.52	− 5.82	13.77	0.474	− 3.39	− 10.42	4.20	0.371
Psychiatric admissions												
All	2.97	− 2.62	8.88	0.304	6.16	1.26	11.30	0.013	− 3.57	− 10.23	3.59	0.319
Children	− 1.84	− 10.77	7.99	0.703	− 2.01	− 8.52	4.96	0.563	− 2.96	− 9.97	4.60	0.432
Adolescent	3.57	− 4.30	12.09	0.384	4.84	− 0.94	10.96	0.102	− 1.60	− 8.36	5.65	0.657
Length of Inpatient stay												
All	6.98	6.74	7.24	0.000	5.58	5.29	5.89	0.000	7.51	7.03	7.99	0.000
Children	1.53	1.19	1.87	0.000	0.59	0.22	0.96	0.002	2.61	1.82	3.40	0.000
Adolescent	10.74	10.34	11.14	0.000	10.87	10.37	11.39	0.000	9.56	8.93	10.19	0.000
Age at admission												
All	6.63	0.66	12.96	0.029	5.18	− 0.51	11.20	0.075	7.68	1.29	14.49	0.018
Children	7.65	0.64	15.14	0.032	6.17	− 0.58	13.39	0.074	8.84	1.55	16.66	0.017
Adolescent	0.10	− 4.67	5.11	0.967	0.05	− 4.71	5.04	0.985	0.06	− 4.75	5.11	0.983

*APC* Annual Percent Change/100.000 residents; *CI* Confidece Interval; i*nf* inferior; *sup* superior

The incidence trend of admissions for Neurological and Psychiatric diagnosis, by sex and age (Table 2) evidenced a general significant decrease for Neurologic diagnosis (APC = − 26.44), particularly evident among children (APC = − 11.10), both male (APC = − 17.62) and female (APC = − 11.89). A significant increase of psychiatric diagnosis among females must be highlighted (APC = 6.16).

The further analyses in Table 1 show a significant global increase of days of inpatients stay (APC = 6.98), significant both for children (APC = 1.53) and adolescent (APC = 10.74) and a significant increase of the age at admission (APC = 6.63).

### Trends in diagnosis

**Fig. 1:**
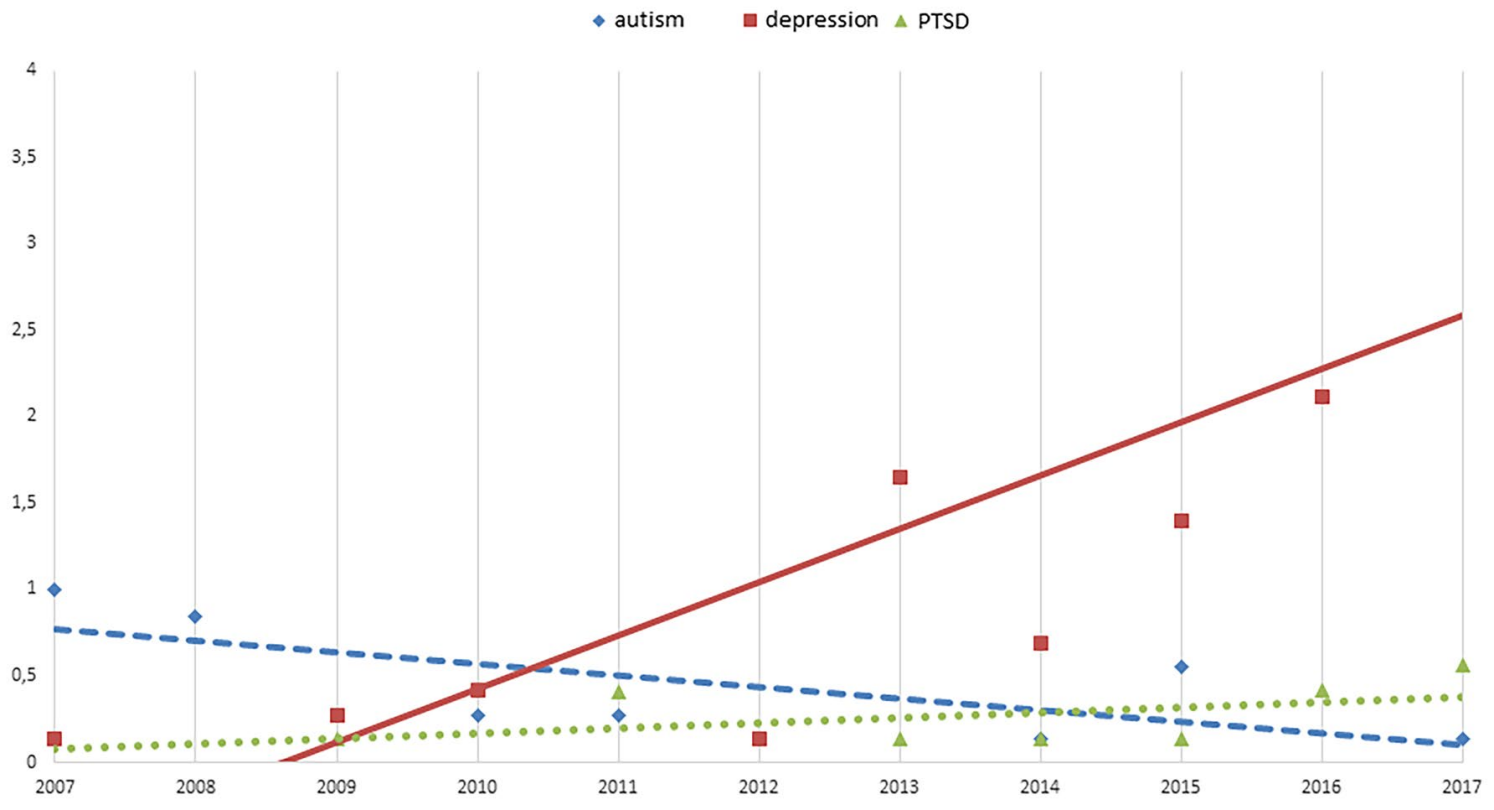
The figure represents the significant trend for the psychiatric diagnosis of Autism (rhombus), Depression (squares), and PTSD (triangles) in the period 2007–2017

Analysis by diagnosis (Table 3; Fig. 1) showed a significant increase in the incidence of depression (APC = 41.89) in particular among females (APC = 40.38). Adolescents (APC = 22.58), in particular female adolescents (APC = 41.30), affected by depressive disorders displayed the greatest increase of inpatients admissions.

**Table 3:** Significant APC values for Psychiatric diagnoses

Psychiatric	Total				Females				Males			
	APC	95% CI inf	95% CI sup	*p*	APC	95% CI inf	95% CI sup	*p*	APC	95% CI inf	95% CI sup	*p*
Autism												
All	− 14.80	− 30.69	− 6.83	0.003	0.00	− 85.91	609.91	1.000	− 9.94	− 21.62	3.48	0.139
Children	− 19.71	− 33.27	− 3.39	0.020	− 50.53	− 82.35	38.63	0.181	− 13.81	− 28.85	4.40	0.128
Adolescent	2.94	− 20.91	33.99	0.829	**	**	**	**	2.94	− 20.91	33.99	0.829
Depression												
All	41.89	27.67	57.97	0.000	40.38	25.02	57.62	0.000	21.65	− 5.51	56.65	0.128
Children	8.18	− 10.89	31.33	0.420	− 2.45	− 23.29	24.05	0.839	3.93	− 9.54	19.36	0.578
Adolescent	22.58	10.03	36.57	0.000	41.30	23.35	61.86	0.000	46.83	− 39.09	254.03	0.392
PTSD												
All	29.41	6.09	57.86	0.010	12.67	− 10.12	41.25	0.300	0.00	− 35.48	55.00	1.000
Children	− 0.30	− 16.25	18.83	0.983	11.66	− 19.20	54.32	0.503	− 0.16	− 17.76	21.20	0.987
Adolescent	20.00	− 20.35	80.82	0.383	20.00	− 20.35	80.82	0.383	**	**	**	**

*APC* Annual Percent Change/100.000 residents; *CI* Confidece Interval; *inf* inferior; *sup* superior

**Data not available due to the low number available per year

We can highlight also the increasing trend for PTSD (APC = 29.41) inpatients admissions and the decreasing trend for Autism (APC = − 14.80) inpatients admissions.

**Fig. 2:**
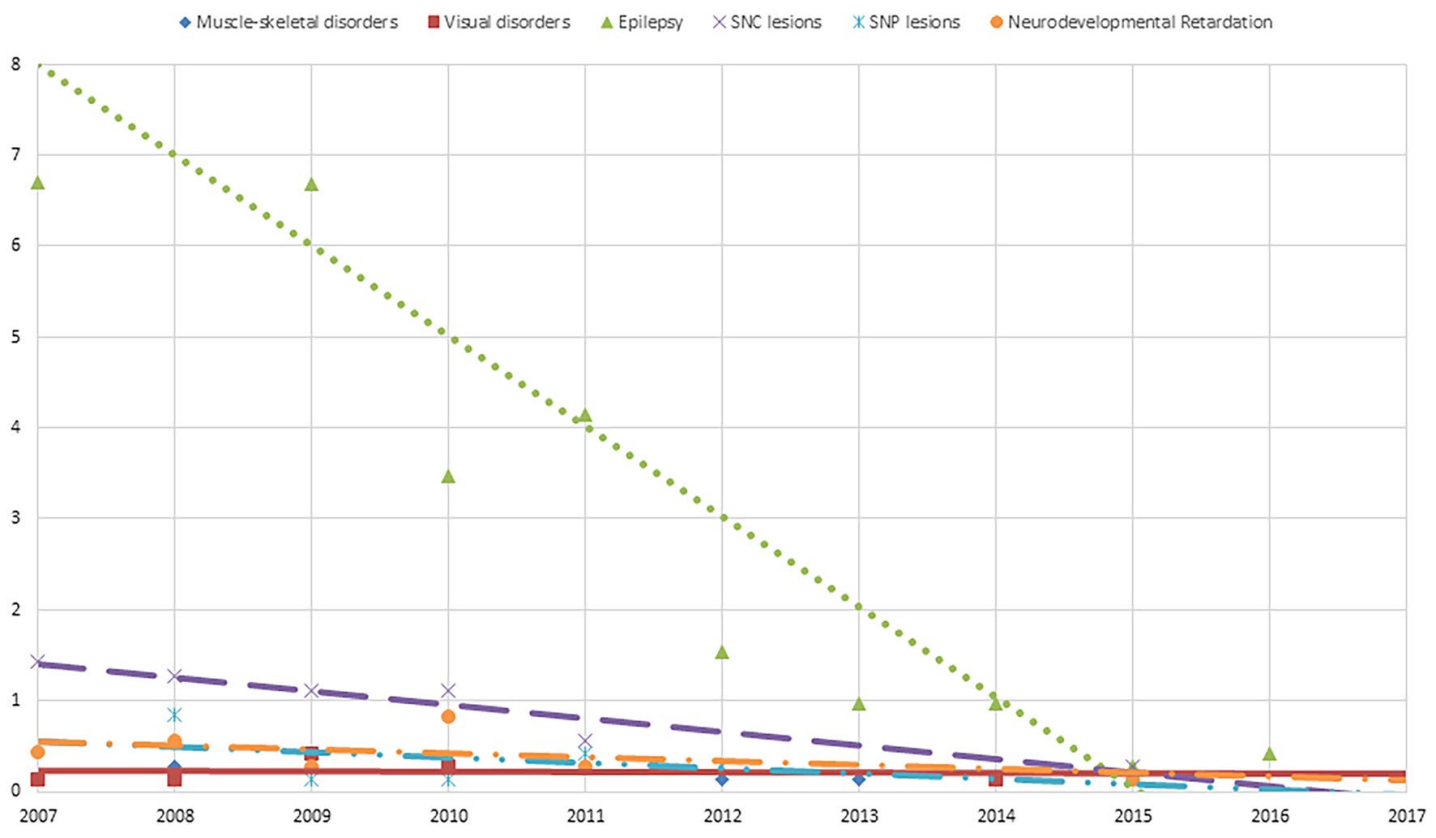
The figure represents the significant trend for the neurological diagnosis of Muscle-skeletal (rhombus), Visual disorders (squares), Epilepsy (triangles), CNS Injuries (X), PNS Injuries (pointed X), Neurodevelopmental Disorders (circle) in the period 2007–2017

Among neurological diagnoses, a significant decrease was evidenced for Neurodevelopmental Disorders (APC = − 27.17), CNS Injuries (APC = − 16.16), PNS Injuries (APC = − 15.63), and Muscle-skeletal disorders (APC = − 26.01), adolescence Epilepsy (APC = -26.66) and CNS Injuries (APC = − 32.61), and child Visual System Disorders (APC = − 25.17) (Table 4; Fig. 2).

**Table 4:** Significant APC values for Neurological diagnoses

Neurologic	Total				Females				Males			
	APC	95% CI inf	95% CI sup	*p*	APC	95% CI inf	95% CI sup	*p*	APC	95% CI inf	95% CI sup	*p*
Muscle-skeletal Disorders**												
All	− 26.01	− 45.06	− 0.36	0.047	0.00	− 31.91	46.87	1.000	100.00	− 81.86	2105.64	0.571
Children	− 6.87	− 33.05	29.55	0.672	0.03	− 15.20	17.98	0.998	101.71	− 31.17	491.18	0.201
Visual Disorders**												
All	**	**	**	**	**	**	**	**	**	**	**	**
Children	− 25.17	− 43.11	− 1.57	0.038	− 1.99	− 47.86	84.20	0.950	− 0.63	− 99.52	20,477.31	0.998
Epilepsy												
All	− 4.44	− 29.28	29.13	0.767	− 3.10	− 31.85	37.80	0.860	0.67	− 70.65	245.29	0.992
Children	**	**	**	**	**	**	**	**	**	**	**	**
Adolescent	− 26.66	− 36.26	− 15.61	0.000	− 23.60	− 33.22	− 12.60	0.000	− 28.06	− 38.21	− 16.24	0.000
CNS Injuries												
All	− 16.16	− 22.25	− 9.59	0.000	− 9.96	− 15.06	− 4.56	0.000	− 13.63	− 20.00	− 6.75	0.000
Children	− 1.38	− 12.44	11.07	0.818	14.87	− 8.82	44.71	0.239	− 7.88	− 16.58	1.73	0.105
Adolescent	− 32.61	− 41.79	− 21.99	0.000	− 15.00	− 31.89	6.08	0.150	− 19.98	− 35.36	− 0.95	0.040
PNS Injuries**												
All	− 15.63	− 27.68	− 1.58	0.030	− 14.87	− 32.19	6.87	0.165	− 16.26	− 32.08	3.25	0.096
Children	− 29.28	− 78.70	134.82	0.571	**	**	**	**	**	**	**	**
Neurodevelopment Disorders**												
All	− 24.44	− 37.07	− 8.51	0.004	− 7.70	− 57.94	102.58	0.841	− 6.13	− 24.06	16.01	0.557
Children	− 11.45	− 28.16	9.13	0.253	0.30	− 40.32	68.57	0.991	− 6.13	− 24.06	16.01	0.557

*APC* Annual Percent Change/100.000 residents; *CI* Confidece Interval; *inf*  inferior; *sup* superior

**Data not available for Adolescent due to the low number of admissions per year

## Discussion

The results of these analyses indicate that major changes have occurred in the utilization of child neuropsychiatric inpatient services over the past 10 years at a large paediatric hospital that served as the only inpatient service for an area with about 4.5 million people. The data are not an effective indicator of the total incidence of child neuropsychiatric disorders in the community, but they represent valid indicator of those requiring inpatient treatment in a specialized psychiatric and neurologic (neuropsychiatric) service in this region due to disorders’ severity or to the inability of the territorial services to manage it in security.

Hospitalizations for psychiatric disorders have significantly increased, and, in parallel, those for neurological conditions have decreased. Patient age and length of stay in the hospital have significantly increased.

A necessary premise in the interpretation of these data is that care for paediatric neurologic and psychiatric disorders is managed in Italy as a unitary medical discipline called child neuropsychiatry. Clinicians are trained in both child neurologic and psychiatric disorders, and neuropsychiatric services, including inpatient, outpatient and day-hospital, are addressed to both categories of disorders.

The first evidence obtained by the data analysis is that the age at admission is progressively increasing from the beginning (2007) to the end (2017) of the observation in favour of adolescent admissions with respect to children admissions. This probably doesn’t mean that the new infant and children cohorts evidence a decrease in neuropsychiatric morbidity, since literature suggests a global increase of neurological and psychiatric disorders in infancy [26].

As a consequence of the priority rules for hospitalization the mean age of inpatient admissions has been probably influenced by the need to contain disruptive or self-damaging behaviours which are highly prevalent in the adolescent population [2].

The increasing proportion of adolescents’ inpatient admissions accounts for the significant decrease in the number of hospitalizations for younger children. Nevertheless, the number of hospitalized adolescents did not increase significantly. It is the increase in the length of hospitalization, rather than the number of adolescent hospitalizations, that accounted for the changes in inpatient service composition. Among the possible factors contributing to this increase in the length of hospitalization, there may be the severity of adolescent psychopathology and the difficulty to arrange for outpatient treatment [5].

The first hypothesis could be supported by the increasing rate of depression, self-damaging or life-threatening acts in adolescent general population evidenced by literature [5, 8]. In our sample the number of inpatient treatments for depression was significantly higher in the last period (2012–2017) with respect to the previous one (2007–2011). This may suggest that the depressive psychopathology is increasing in severity and it needs longer periods of treatment with respect to the past.

The second may be related to a possible inadequacy of the neuropsychiatric outpatient services, whose resources or organization may not be completely updated with respect to the containment of the rising severe adolescent psychopathology [27]. On the other hand, it may be also related to the family difficulty in the management of the adolescents expressing psychopathology. As mentioned in the introduction the changes in social and relational dynamics with a reduction of the role of authority in the familial relationships may have contributed to a difficulty of parents in establishing relations of authority and hierarchy [16] to contain the adolescent. Moreover, the family is often unprepared to face with the new forms of psychopathological expression of the adolescents, related or not to the new technologies. This may account for the clinicians’ difficulty in organizing secure hospital discharge into the origin family, and give reason of the recently underlined overuse of community treatment in Italian reality [28].

Another evidence is the progressive gender differentiation, with an increase of female inpatient admissions. This underlines that the depressed female adolescents progressively occupied the inpatient service, excluding the children and also male adolescents. Moreover, female adolescents generally display a higher need for long inpatient treatments. Based on the available data only speculation is possible. The largest diagnostic group in our sample is represented by the eating disorders that are prevalent in female gender (*n* = 216, 91%) and request long periods of inpatient treatment [9]. Nevertheless, the diagnosis of eating disorders do not significantly increase in the observed period. It is most possible that the quality of female depression is different from that of male subjects, thus requiring longer inpatient stay. Female sex is generally more prone to depression with respect to the male one [6, 7], and also it more frequently displays personality features which indicate a liability towards depressive disorders [29]. Nevertheless, the relative increase in frequency and length of depressive episodes in female adolescents in our sample may be also due also to social and environmental changes of contemporary society: the high prevalence and in some cases the increase of physical or sexual abuse [30], stalking and sexting [31], bullying [32], and prostitution [33]. Moreover, when the abuses are intra-familial or strictly related to the social environment they may represent also a severe obstacle in organizing the demission at home [2, 34].

The final evidence regards the reduction of inpatient treatments for neurological disorders with respect to psychiatric disorders. The authors don’t believe that this means an absolute reduction of neurologic disease, that literature estimates in growth [26]. It is very possible that the subjects with neurological problems have been admitted to paediatric wards because they are more easily manageable without a specific organization and structural containment. Moreover, it is possible that clinicians estimated dangerous to admit in the same ward agitated and disruptive adolescents with infants and young children affected with neurological disorders. Of fact, the evidence suggests a transition of the neuropsychiatric ward to a more strictly psychiatric function in the last few years.

### Clinical implications

As claimed by many authors on large population studies [5–7] an increase of depressive problems in adolescents’ population emerges also in the trend of inpatient’s admission of the present research. In particular adolescent female subjects with severe depressive symptoms and a tendency to a long-lasting stay in inpatient service has become the greatest users of neuropsychiatric inpatient service. The organization of neuropsychiatric services is called to adequate the services for the early recognition and treatment [5, 35] to avoid chronic course of the disease and its negative existential outcomes [36].

As it concerns the Italian situation, it is evident that the progressive replacement of neurologic patients in the inpatient service poses the question of where the children affected with neurologic diseases needing for inpatient treatments are cared. In the present hospital experience they have been located in general paediatric inpatient services where neuropsychiatrists manage them with consultations. This suggests the need for an overall higher number of beds (and clinicians) dedicated to neurologic patients as suggested by international authors [5].

The long-lasting stay into the inpatient service remarks for another potential field of intervention. Johnson and coworkers [37] have pointed out that adequate parenting behaviours are influential on the resilience of the children, allowing better stress management and a better adjustment in adulthood. Family interventions should be strategically important to reduce untoward outcomes[2]. A particular emphasis must be given to the need for proper training of family doctors and paediatricians for early diagnose and treatment of neuropsychiatric problems in adolescence, to the management of relational intra-family problems, and for offering support for parenting [38]. The proper post-demission management may represent a substantial factor to reduce the serious consequences that the youth psychopathology can generate in terms of rising mental suffering and costs for healthcare systems [28].

Finally, it needs to be underlined that depressive psychopathology is more prevalent in females starting with puberty, and may require long inpatients treatment in case of significant suicide risk. More research should be addressed to understand the reasons of this emergence in the social context, and adequate preventive interventions on the population should be performed. Moreover they should be more specifically explored the most cost-effective treatments to address adolescent subjects, possibly applying those who were demonstrated as effective in adults and testing those who best fit to adolescent population [39], but even exploring new specific methods studied for adolescent population [40].

### Limitation of the study

The present study presents some limitations. First, the amount of data presented for the study is limited because they were collected in the database of the paediatric hospital for administrative purposes and not organized for research aims.

Second, even though the catchment area for the paediatric hospital that provided the data was the entire Piedmont region, it is still possible that neuropsychiatric inpatient treatments for less severe psychiatric or neurologic disorders were provided in paediatric inpatients services at peripherals hospitals. Thus, the data here presented may not necessarily constitute all the neuropsychiatric inpatient treatments.

Third, even though PIVA is a large region, the trend expressed by this research may not be generalizable to other regions of Italy.

Fourth, data about inpatient treatment of neurological/neuropsychiatric subjects admitted to paediatric services without a passage through the emergency service of our hospital are not available since the admission/demission diagnosis may not reflect the neuropsychiatric one.

Fifth, it is possible that some of the older adolescents with severe aggressive behaviours not manageable in an adolescent unit might have been hospitalized in adult units.

### Conclusion

In conclusion, from our data it emerges a significant increasing trend in the use of our neuropsychiatric inpatients service for the treatment of female adolescents affected with depressive disorders, and requiring long inpatients stay. The inpatient neuropsychiatric service of an Italian region with the same population of Ireland is no more available for inpatient treatment of children affected with neurological disorders. A re-organization and a significant increase of the inpatient beds for neurological and psychiatric treatments available in this region, and possibly also in other European regions, is needed to accomplish to the increasing needs of care of child and adolescent population [2]. As for suicide prevention [41], individual self-driven and social driven therapeutic prevention programmes for depression in female adolescents should be adopted. Greater support to the families for at-home management of their suffering children may also be needed to reduce the number and the length of inpatients treatments.

The present study may represent a starting point for a collaboration with other neuropsychiatry units in other parts of Italy and can be useful for future comparisons with other child neuropsychiatry facilities.

## Data Availability

Data can be made available upon request. However, sharing of the data may require approval and some access restrictions may apply. Requests may be sent to the corresponding author.

## Code availability

Custom code.

## References

[1] McCann TV, Lubman DI (2012). Young people with depression and their experience accessing an enhanced primary care service for youth with emerging mental health problems: a qualitative study. BMC Psychiatry.

[2] Smorti M, Milone A, Gonzalez Gonzalez J, Vitali Rosati G (2019). Adolescent selfie: an Italian Society of Paediatrics survey of the lifestyle of teenagers. Ital J Pediatr.

[3] Maughan B, Stafford M, Shah I, Kuh D (2014). Adolescent conduct problems and premature mortality: follow-up to age 65 years in a national birth cohort. Psychol Med.

[4] Davis JP, Pedersen ER, Tucker JS, Dunbar MS, Seelam R, Shih R, D'Amico EJ (2019). Long-term associations between substance use-related media exposure, descriptive norms, and alcohol use from adolescence to young adulthood. J Youth Adolesc.

[5] Mojtabai R, Olfson M, Han B (2016). National trends in the prevalence and treatment of depression in adolescents and young adults. Pediatrics.

[6] Patalay P, Gage SH (2019). Changes in millennial adolescent mental health and health-related behaviours over 10 years: a population cohort comparison study. Int J Epidemiol.

[7] Townsend L, Musci R, Stuart E (2019). Gender differences in depression literacy and stigma after a randomized controlled evaluation of a universal depression education program. J Adolesc Health.

[8] Ulberg R, Hersoug AG, Høglend P (2012). Treatment of adolescents with depression: The effect of transference interventions in a randomized controlled study of dynamic psychotherapy. Trials.

[9] Demmler JC, Brophy ST, Marchant A (2020). Shining the light on eating disorders, incidence, prognosis and profiling of patients in primary and secondary care: National data linkage study. Br J Psychiatry.

[10] Clarke G, Harvey AG (2012). The complex role of sleep in adolescent depression. Child Adolesc Psychiatr Clin N Am.

[11] Sarchiapone M, Mandelli L, Carli V (2014). Hours of sleep in adolescents and its association with anxiety, emotional concerns, and suicidal ideation. Sleep Med.

[12] Najolia GM, Buckner JD, Cohen AS (2012). Cannabis use and schizotypy: the role of social anxiety and other negative affective states. Psychiatry Res.

[13] Amianto F, Spalatro AV, Rainis M (2018). Childhood emotional abuse and neglect in obese patients with and without binge eating disorder: personality and psychopathology correlates in adulthood. Psychiatry Res.

[14] Sciberras E, Ohan J, Anderson V (2012). Bullying and peer victimisation in adolescent girls with attention-deficit/hyperactivity disorder. Child Psychiatry Hum Dev.

[15] Soler L, Segura A, Kirchner T, Forns M (2013). Polyvictimization and risk for suicidal phenomena in a community sample of spanish adolescents. Violence Vict.

[16] Perers C, Bäckström B, Johansson BJ, Rask O (2021). Methods and strategies for reducing seclusion and restraint in child and adolescent psychiatric inpatient care. Psychiatr Q.

[17] Bauman Z (2011). Culture in a liquid modern world.

[18] Amianto F, Daga GA, Bertorello A, Fassino S (2013). Exploring personality clusters among parents of ED subjects. Relationship with parents’ psychopathology, attachment, and family dynamics. Compr Psychiatry.

[19] Silva TC, Larm P, Vitaro F (2012). The association between maltreatment in childhood and criminal convictions to age 24: a prospective study of a community sample of males from disadvantaged neighbourhoods. Eur Child Adolesc Psychiatry.

[20] Erdelja S, Vokal P, Bolfan M (2013). Delinquency in incarcerated male adolescents is associated with single parenthood, exposure to more violence at home and in the community, and poorer self-image. Croat Med J.

[21] Xu J, Shen L-x, Yan C-h (2014). Parent-adolescent interaction and risk of adolescent internet addiction: a population-based study in Shanghai. BMC Psychiatry.

[22] Rousseau S, Vanderfaeillie J, Desoete A (2014). The distinction of “psychosomatogenic family types” based on parents’ self reported questionnaire information: a cluster analysis. Fam Syst Heal.

[23] Fassino S, Amianto F, Abbate-Daga G (2009). The dynamic relationship of parental personality traits with the personality and psychopathology traits of anorectic and bulimic daughters. Compr Psychiatry.

[24] Regione Piemonte. PiSta - Piemonte STAtistica e B.D.D.E. http://www.ruparpiemonte.it/infostat/index.jsp

[25] Clegg LX, Hankey BF, Tiwari R (2009). Estimating average annual per cent change in trend analysis. Stat Med.

[26] Whiteford HA, Ferrari AJ, Degenhardt L, Feigin V, Vos T (2015). The global burden of mental, neurological and substance use disorders: an analysis from the global burden of disease study 2010. PLoS ONE.

[27] Lee TK, Wickrama KAS, O’Neal CW, Lorenz FO (2017). Social stratification of general psychopathology trajectories and young adult social outcomes: a second-order growth mixture analysis over the early life course. J Affect Disord.

[28] Barbui C, Papola D, Saraceno B (2018). Forty years without mental hospitals in Italy. Int J Ment Health Syst.

[29] Delvecchio G, Garzitto M, Fagnani C (2016). Normative data and effects of age and gender on temperament and character dimensions across the lifespan in an Italian population: a cross-sectional validation study. J Affect Disord.

[30] Taquette SR, Leite D, Monteiro M (2019). Causes and consequences of adolescent dating violence: a systematic review. J Inj Violence Res.

[31] Breiding MJ, Smith SG, Basile KC, Walters ML, Chen J, Merrick MT (2014). Prevalence and characteristics of sexual violence, stalking, and intimate partner violence victimization–national intimate partner and sexual violence survey, United States, 2011. MMWR Surveill Summ.

[32] Williams SG, Godfrey AJ (2011). What is cyberbullying and how can psychiatric mental health nurses recognize it?. J Psychosoc Nurs Ment Health Serv.

[33] Fredlund C, Svensson F, Svedin CG (2013). Adolescents’ lifetime experience of selling sex: development over five years. J Child Sex Abus.

[34] Sonu S, Post S, Feinglass J (2019). Adverse childhood experiences and the onset of chronic disease in young adulthood. Prev Med (Baltim).

[35] Signorini G, Singh SP, Boricevic-Marsanic V (2017). Architecture and functioning of child and adolescent mental health services: a 28-country survey in Europe. The Lancet Psychiatry.

[36] Sellers R, Warne N, Pickles A (2019). Cross-cohort change in adolescent outcomes for children with mental health problems. J Child Psychol Psychiatry Allied Discip.

[37] Johnson JG, Liu L, Cohen P (2011). Parenting behaviours associated with the development of adaptive and maladaptive offspring personality traits. Can J Psychiatry.

[38] Amianto F, Bertorello A, Spalatro A (2014). Adlerian parental counseling in eating disorders: preliminary data of a controlled clinical trial. Eat Weight Disord.

[39] Nararro-Haro MV, Hoffman HG, Garcia-Palacios A (2016). The use of virtual reality to facilitate mindfulness skills training in dialectical behavioral therapy for borderline personality disorder: a case study. Front Psychol.

[40] Beckstead DJ, Lambert MJ, DuBose AP, Linehan M (2015). Dialectical behavior therapy with American Indian/Alaska Native adolescents diagnosed with substance use disorders: Combining an evidence based treatment with cultural, traditional, and spiritual beliefs. Addict Behav.

[41] Iyengar U, Snowden N, Asarnow JR, Moran P, Tranah T, Ougrin D (2018). A further look at therapeutic interventions for suicide attempts and self-harm in adolescents: an updated systematic review of randomized controlled trials. Front Psychiatry.

